# Open-Cavity Spun Fiber Raman Lasers with Dual Polarization Output

**DOI:** 10.1038/s41598-017-13193-7

**Published:** 2017-10-20

**Authors:** Javier Nuño, Giuseppe Rizzelli, Francesca Gallazzi, Francisco Prieto, Concepción Pulido, Pedro Corredera, Stefan Wabnitz, Juan D. Ania-Castanon

**Affiliations:** 1 Instituto De Óptica’ Daza De Valdes’, IO-CSIC, Madrid, 28006 Spain; 2 Saint Louis University - Madrid Campus, Madrid, 28003 Spain; 3 Instituto de Estructura de la Materia, IEM-CSIC, Madrid, 28006 Spain; 4 INO-CNR and Università degli Studi di Brescia, Dipartimento di Ingegneria dell’ Informazione, Brescia, 25123 Italy; 5 Novosibirsk State University, 1 Pirogova str, Novosibirsk, 630090 Russia; 6 Departamento de Electrónica, Universidad de Alcalà, Escuela Politécnica Superior, 28871 Madrid, Spain

## Abstract

Random distributed feedback fiber Raman lasers, where the feedback mechanism is provided by Rayleigh backscattering, have attracted a good deal of attention since they were first introduced in 2010. Their simple and flexible design, combined with good lasing efficiency and beam quality properties, comparable to those of standard cavity lasers, have led to multiple applications, particularly in the fields of fiber sensing and optical communications. In spite of these advances, the polarization properties of random fiber Raman lasers, which can strongly affect their performance in both sensing and communications, have barely been explored so far. In this article we experimentally and theoretically study the polarization properties of different open-cavity laser designs, based on either standard transmission fibers or low polarization-mode-dispersion spun fibers. By using high-power, highly-polarized pumps, we demonstrate controllable polarization-pulling and simultaneous lasing at close wavelengths with different output polarization properties in random distributed feedback fiber Raman lasers. These results advance our understanding of the polarization dynamics in ultralong lasers, and pave the way to the design of novel fiber laser sources capable of polarization-sensitive sensing and distributed amplification.

## Introduction

Random lasing exploits the multiple scattering of photons in a disordered gain medium, a mechanism which may provide a coherent light source without a traditional cavity^1^. Recently, random lasing was demonstrated in standard telecommunication optical fibers (SMFs)^2,3^. At first, random lasing was obtained as the asymptotic operating regime of extremely long cavity lasers^4^. Next, random laser configurations where developed involving arbitrary lengths of fiber, by taking advantage of the distributed feedback mechanism provided by Rayleigh backscattering^5^. These random distributed feedback fiber lasers (RDFLs) offer narrow spectrum generation, quasi-CW operation, and the same high beam quality as normally expected from a single-mode fiber laser. In addition, unlike other kinds of random lasers, RDFLs also exhibit excellent pump energy conversion efficiencies, which are comparable to those of conventional cavity lasers^6–10^. These qualities, coupled with their simple design, made RDFLs an excellent solution for a large number of applications, ranging from the interrogation of remote and quasi-distributed sensors, to distributed amplification in optical communication links, and in distributed sensing schemes.

In spite of all of this progress, quite remarkably the study of the polarization properties of RDFLs, and the management of the evolution of the state of polarization (SOP) of light in these sources still remains as a pending challenge. Effects such as polarization dependent gain (PDG) and polarization mode dispersion (PMD) pose important limitations to the performance of next-generation transparent optical communication systems (which rely on distributed amplification and nonlinearity mitigation). Additionally, PDG also sets important constraints to the design of low-noise fiber lasers, since their PDG significantly contributes to relative intensity-noise (RIN) fluctuations^11,12^. Finally, polarization control is critical for applications of these sources in some sensing techniques^13^. In this context, polarization pulling through nonlinear effects has been demonstrated as a key enabling technology to achieve polarization control in fiber optics photonic devices^14–18^. Thanks to PDG, Raman polarizers^16^ are devices capable of producing a highly polarized amplified output. In order to achieve efficient polarization pulling coupled with high gain, Raman polarizers require a highly polarized high-power pump, and a sufficiently long span of low-PMD fiber. One of such fiber is the so-called “spun” optical fiber, which is produced by quickly rotating (or oscillating) the fiber preform during fiber drawing. This results in the averaging of fiber core non-uniformities, which effectively cancels out the total fiber birefringence. Therefore, the resulting spun fiber exhibits a negligible value of the average PMD^19,20^. The main advantage of spun low-birefringence fibers is that, unlike traditional polarization-maintaining fibers, they preserve any light SOP, and not only linear polarizations. In particular, spun fibers are able to maintain circular polarization states. Moreover, when fabricated by using standard fiber preforms, spun fibers exhibit similar transmission characteristics (dispersion, attenuation, nonlinearity) to corresponding transmission fibers, and can be potentially produced at a relatively low cost. Thanks to their very low PMD values, spun fiber are particularly suited for implementing devices based on polarization attraction, since they allow for improved polarization alignment between pump and signal, and consequently lead to high Raman gain values.

The emission of a polarized output from a random laser was observed in a number of active media^21–23^. Moreover, it is well-known that Raman gain in fibers is highly polarization dependent^24^, since perpendicular SOPs for pump and signal result in negligible signal amplification. Consequently, the efficiency of RDFL can be expected to be significantly improved by the possibility of imposing an appropriate polarization control. Recently, different configurations were proposed to produce a highly polarized output from RDFLs. The first attempt involved using a polarized pump in a 50-km long standard fiber cavity, and produced a partially polarized output radiation^25^, with a lasing threshold that was strongly dependent on the pump SOP. Subsequently, the use of a polarization-maintaining (PM) fiber^26^ has led to a linearly polarized output, however with a relatively low pumping efficiency. Finally, a PM-fiber based random laser^27^, pumped with linearly polarized light, was used to demonstrate a linearly polarized output with a close-to-ultimate conversion efficiency, even in the case of high-order random Raman lasing^28^.

In this article we propose, theoretically analyze and experimentally prove a different approach to achieve polarization control coupled with high conversion efficiency in RDFLs. Our method is based on the use of highly polarized pumps in an open-cavity (single-grating) RDFL configuration, and the concept of efficient Raman polarization pulling in a low-PMD fiber. We study the impact of PMD on overall RDFL performance and intra-cavity polarization pulling, by comparing the bi-directional output from active cavities composed of either SMF or a specially designed low-PMD, SMF-based, rapidly spun fiber. Furthermore, we present what is, to our knowledge, the first demonstration of simultaneous random distributed feedback lasing from the same source with different output polarization properties at different laser wavelengths. These results help advancing our understanding of the polarization dynamics in ultralong fiber lasers, and pave the way to the design of novel fiber laser sources with potential applications in areas such as polarization-sensitive sensing or polarization-selective distributed amplification.

## Results

### Experimental setup

**Figure 1:**
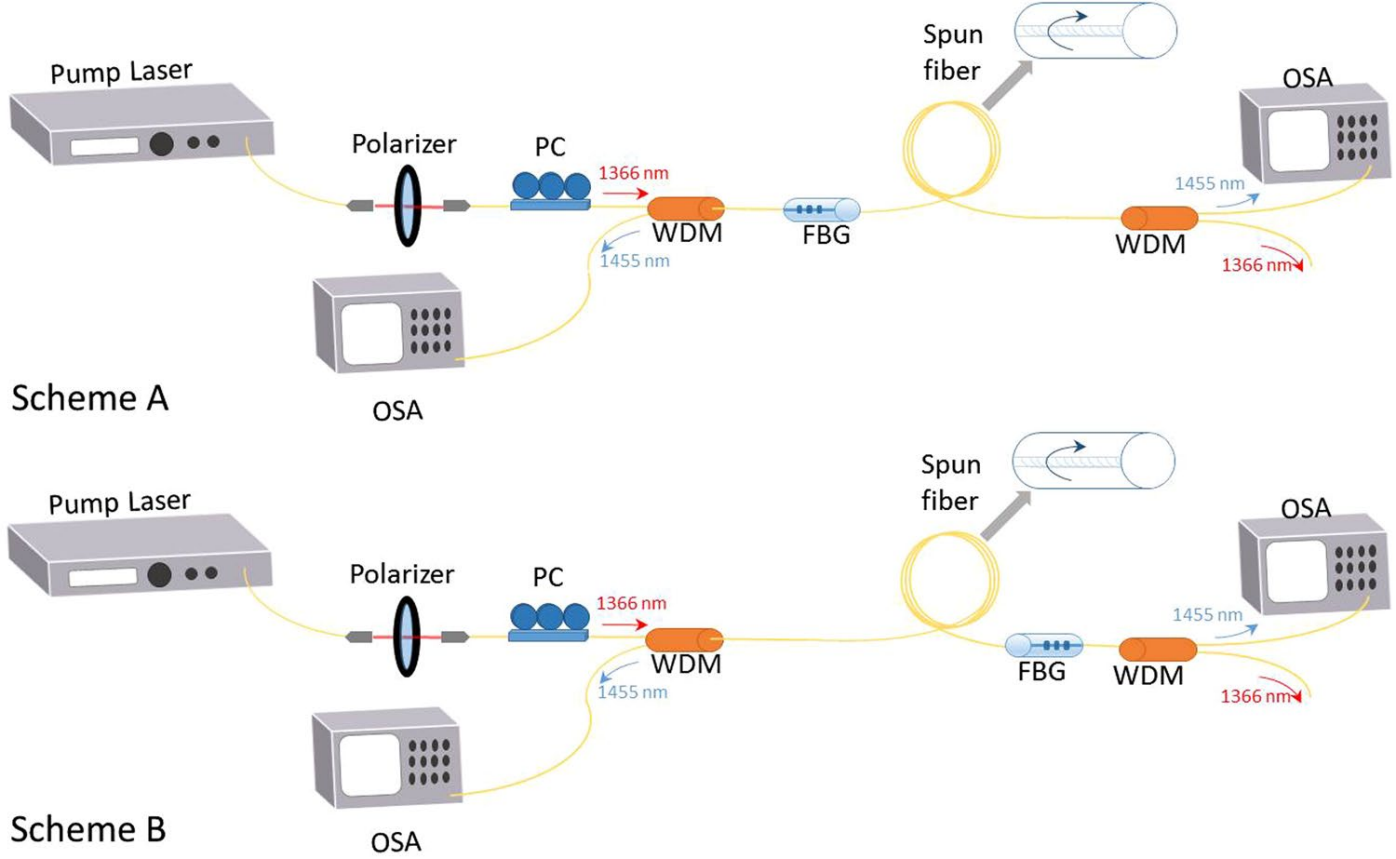
Schematic of the experimental setup for an open cavity laser. PC: Polarization controller. WDM: Wavelength division multiplexer. FBG: Fiber Bragg grating. OSA: Optical spectrum analyzer. In Scheme A or 1, with a single FBG at the pump input and, in Scheme B or 2 at the output end.

The schematic diagrams of the single-grating open cavity lasers that we designed for our experiments are depicted in Fig. 1. In both cases, the pump source is a CW fiber laser source at 1366 nm, able to produce up to 38 dBm. As it is expected in a fiber laser source, the output is naturally depolarized, exhibiting a degree of polarization (DOP) of less than 10%. A polarized pump beam (in excess of 99%) was thus achieved by means of a polarizing prism, after which the polarized output was injected into a fiber polarization controller (PC), which can be used to generate a range of different SOP values.

**Figure 2:**
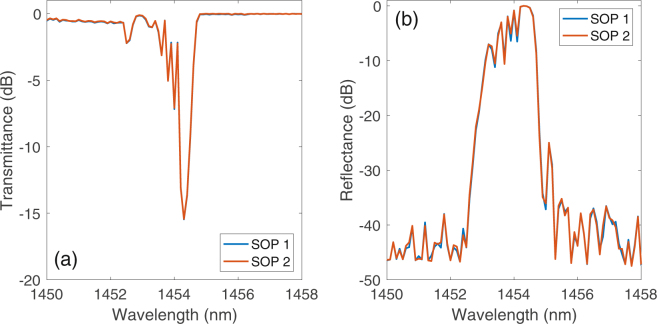
(**a**) FBG transmittance and (**b**) reflectance for two orthogonal SOPs.

As shown in Fig. 1, the output pump from the PC was injected through a WDM into the open-cavity laser, which has a single high reflectivity (close to 90%) fiber Bragg grating (FBG), centered at 1454.5 nm, on either the input or the output pumping end, depending on the chosen design. The transmittance and reflectance of this FBG do not display a measurable dependence on the polarization of the incident signal. In Fig. 2 the obtained transmittance and reflectance characteristics for two different orthogonal SOPs are shown. As with all similar open-cavity designs, Rayleigh backscattering provides the random distributed feedback mechanism for the open side of the cavity. The 2 km spun fiber was extruded from a standard fiber preform, and its main characteristics are summarized in Table 1.

**Table 1:** Parameters of the employed fibers.

Fiber type	Spun	SMF
Core diameter	9.5 *μ*m	8.2 *μ*m
Fiber diameter	125 *μ*m	125 *μ*m
Coating diameter	248 *μ*m	243 *μ*m
Core numerical aperture	0.12	0.14
MFD at 1550 nm	10.4 *μ*m	10.4 *μ*m
Background loss at 1550 nm	0.43 dB/km	0.2 dB/km
Spinning period	<1 cm	—
Beat length	>1.8 m	—

### Theoretical model

In the proposed open cavity RDFL configuration of Fig. 1, there are three main propagating CW beams interacting with each other: the forward pump at 1366 nm, the signal co-propagating with it, and the counter-propagating signal. The additional, low-power Rayleigh-backscattered 1366 nm component which is counter-propagating with respect to the pump may be generally ignored, as it only plays a limited role in the signal amplification process. By considering the most basic three-wave interaction model within a Kerr- and Raman-active medium^16^, with the phenomenological addition of Rayleigh backscattering, one obtains the following equations

1 $$\begin{array}{c}\pm {\partial }_{z}{{\bf{S}}}^{(s\pm )}=-\alpha {{\bf{S}}}^{(s\pm )}+\gamma ({{\bf{S}}}^{(s\pm )}\times {{\bf{J}}}_{s}{{\bf{S}}}^{(s\pm )}+{{\bf{S}}}^{(s\pm )}\times {{\bf{J}}}_{x}^{-}{{\bf{S}}}^{(s\mp )}+{{\bf{S}}}^{(s\pm )}\times {{\bf{J}}}_{x}^{\pm }{{\bf{S}}}^{(p)})\\ \quad \quad \quad \quad +\frac{g}{2}[{S}_{0}^{(p)}{J}_{R0}({{\bf{S}}}^{(s\pm )}+{{\bf{S}}}^{(n\pm )})+({S}_{0}^{(s\pm )}+{S}_{0}^{(n\pm )}){{\bf{J}}}_{R}^{\pm }{{\bf{S}}}^{(p\pm )}]+\varepsilon {{\bf{J}}}_{\varepsilon }{{\bf{S}}}^{(s\mp )}\end{array}$$

2 $$\begin{array}{c}\pm {\partial }_{z}{S}_{0}^{(s\pm )}=-\alpha {S}_{0}^{(s\pm )}+\frac{g}{2}[{S}_{0}^{(p)}{J}_{R0}({S}_{0}^{(s\pm )}+{S}_{0}^{(n\pm )})+{S}_{1}^{(p)}{J}_{R1}({S}_{1}^{(s\pm )}+{S}_{1}^{(n\pm )})\\ \quad \quad \quad \quad +{S}_{2}^{(p)}{J}_{R2}({S}_{2}^{(s\pm )}+{S}_{2}^{(n\pm )})+{S}_{3}^{(p)}{J}_{R3}({S}_{3}^{(s\pm )}+{S}_{3}^{(n\pm )})]+\varepsilon {{\bf{S}}}^{(s\mp )}\end{array}$$

Here, the constant *α* is the fiber linear loss coefficient, *γ* is the nonlinear coefficient, g is the Raman gain, and $\varepsilon$ is the Rayleigh coefficient. ${{\bf{S}}}^{(j)}=({S}_{1}^{(j)},{S}_{2}^{(j)},{S}_{3}^{(j)})$ represent the Stokes vectors of the pump, of the co(counter)-propagating signal, and of the co(counter)-propagating noise for j = p, s + (s−) and n + (n−), respectively. Moreover, the power of the pump and signals is described by ${S}_{0}^{(j)}={(({S}_{1}^{(j)}{)}^{2}+{({S}_{2}^{(j)})}^{2}+{({S}_{3}^{(j)})}^{2})}^{\mathrm{1/2}}$, and the noise ${{\bf{S}}}^{(n\pm )}$ is randomly generated at any point of the fiber, in any component of the corresponding Stokes vector. The total noise power reads as

3 $${S}_{0}^{(n\pm )}=2h{\nu }_{s}{\rm{\Delta }}{\nu }_{s}(1+\frac{1}{{e}^{h({\nu }_{p}-{\nu }_{s})/{k}_{B}T}-1})$$

where h is Plank’s constant, *k*_*B*_ is Boltzmann’s constant, T is the absolute temperature of the fiber, ${\nu }_{{\rm{j}}}$ are the corresponding frequencies of the pump and signals, and ${\rm{\Delta }}{\nu }_{j}$ is the effective bandwidths of the signals.

**J**_***s***_, **J**_***x***_, **J**_***ε***_ and **J**_**r**_ are tensors associated with the presence of self-phase modulation (SPM), cross-phase modulation (XPM), Rayleigh and Raman scattering, respectively. In the case of a spun fiber, when taking into account its relatively short length and low PMD value, the value of all of these tensors can be considered a constant along the longitudinal coordinate of the fiber *z*. By following the analytical approach described in^29^, the effects of SPM can be neglected. Whereas the XPM and Raman tensors can be simplified to ${{\bf{J}}}_{x}^{\pm }=-8/9diag\mathrm{(1},1,\pm \mathrm{1)}$ and ${{\bf{J}}}_{{\rm{R}}}^{\pm }=\mathrm{(2}\pm \mathrm{1)}/3diag\mathrm{(1},1,\pm \mathrm{1)}$, respectively. In an isotropic fiber (i.e., with no linear birefringence), the Rayleigh tensor reads as ${{\bf{J}}}_{{\boldsymbol{\varepsilon }}}=diag\mathrm{(1},1,-\mathrm{1)}$ ^30^. We may assume this value to be also applicable to the case of an ideal spun fiber. Conversely, in modern SMFs with low but random birefringence, the situation is more complex: fiber birefringence affects both the signal and its Rayleigh backscattered counterpart. In this situation, by modeling the SOP evolution as a three-dimensional Brownian motion, and considering that the effective fiber length is much larger than the polarization beat length, it can be shown that when a polarized signal is Rayleigh backscattered, the DOP of the backscattered signal is reduced to only one third of the value of the original signal DOP^30^. Consequently, one may expect to observe a reduction of the signal DOP in SMF based random lasers, when compared with the case of a spun fiber based RDFL.

A set of equations equivalent to Eqs (1–2) can be used for describing the longitudinal evolution of the Stokes vector of the pump and of its power:

4 $$\begin{array}{c}{\partial }_{z}{{\bf{S}}}^{(p)}=-\alpha {{\bf{S}}}^{(p)}+\gamma ({{\bf{S}}}^{(p\pm )}\times {{\bf{J}}}_{p}{{\bf{S}}}^{(p)}+{{\bf{S}}}^{(p)}\times {{\bf{J}}}_{x}^{+}{{\bf{S}}}^{(s+)}+{{\bf{S}}}^{(p)}\times {{\bf{J}}}_{x}^{-}{{\bf{S}}}^{(s-)})\\ \quad \quad \quad \,\,-\frac{g}{2}[({S}_{0}^{(s+)}+{S}_{0}^{(s-)}){J}_{R0}{{\bf{S}}}^{(p)}+{S}_{0}^{(p)}{{\bf{J}}}_{R}^{+}{{\bf{S}}}^{(s+)}+{S}_{0}^{(p)}{{\bf{J}}}_{R}^{-}{{\bf{S}}}^{(s-)}]\end{array}$$

5 $$\begin{array}{c}{\partial }_{z}{S}_{0}^{(p)}=-\alpha {S}_{0}^{(p)}-\frac{g}{2}[{S}_{0}^{(s+)}{J}_{R0}{S}_{0}^{(p)}+{S}_{1}^{(s+)}{J}_{R1}^{+}{S}_{1}^{(p)}+{S}_{2}^{(s+)}{J}_{R2}^{+}{S}_{2}^{(p)}+{S}_{3}^{(s+)}{J}_{R3}^{+}{S}_{3}^{(p)}\\ \quad \quad \quad \,\,+{S}_{0}^{(s-)}{J}_{R0}{S}_{0}^{(p)}+{S}_{1}^{(s-)}{J}_{R1}^{-}{S}_{1}^{(p)}+{S}_{2}^{(s-)}{J}_{R2}^{-}{S}_{2}^{(p)}+{S}_{3}^{(s-)}{J}_{R3}^{-}{S}_{3}^{(p)}]\end{array}$$

However, since the pump power always remains much higher than the signal power, pump depletion and polarization-dependent phase shifts due to the Raman effect and to XPM, respectvely, can be largely neglected. Subsequently, in our simplified model, the Stokes vector of the pump is not affected by the presence of the signal, and the pump power evolution is only affected by the presence of linear attenuation. A more complete theoretical analysis of the process of polarization pulling between pump and signal polarizations in spun fibers can be found in^31–33^.

Based on our modelling Eqs (1–3), we numerically studied both the mirrorless case and the two open-cavity configurations illustrated in Fig. 1: the position of the FBG thus determines the boundary conditions. We used the following physical parameters: *g* = 0.7 *km* ^−1^ · *W* ^−1^, *ε* = 10^−3^ *km* ^−1^, $\gamma \,=1.5$ *km* ^−1^ · *W* ^−1^, a loss coeffcient *α* = 0.5 and 0.35 $dB\cdot k{m}^{-1}$ for the pump and the signal, respectively; the total fiber length was set to be equal to 4 km. In all of the three configurations involving a fully polarized pump, we observed that lasing is more efficient than the case where a depolarized pump is used. This occurs thanks to the higher gain which is experienced by the Stokes component of the signal that is aligned with the polarization axis of the pump. In the mirrorless configuration, our numerics show that the power of the co-propagating signal is higher than the power of the counter-propagating signal. In fact, the modulus of the Raman tensor is three times lower for the counter-propagating case ($|{{\bf{J}}}_{{\bf{R}}}^{+}|=1$ and $|{{\bf{J}}}_{{\bf{R}}}^{-}|=1/3$).

**Figure 3:**
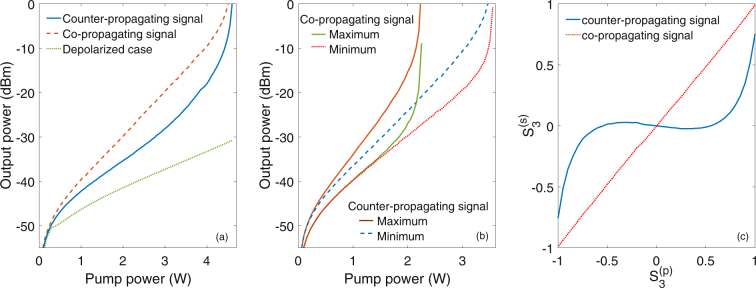
Simulation results: (**a**) Output power as function of the pump power for the mirrorless configuration. (**b**) Maximum and minimum output power for scheme B. (**c**) Output circular polarization components (${S}_{3}^{s\pm }$) as function of the pump circular polarization component (${S}_{3}^{p}$), for a pump power of 1.6 W.

In Fig. 3(a) we illustrate, for the mirrorless configuration, the dependence on pump power of the output laser power from either the co-propagating or the counterpropagating signal. Here the output power in the depolarized pump case is also added, for the ease of comparison. In terms of SOP, our simulations show that the co-propagating signal is fully polarized, with a DOP close to 100%. Conversely, the counter-propagating signal only partially polarized (DOP < 85%), because of the corresponding reduced degree of polarization attraction. Indeed, the SOP of the co-propagating output signal is fully aligned with the SOP of the pump. On the other hand, while the first and the second components of the Stokes vector of the counter-propagating signal are attracted to the same components of the pump Stokes vector, the third (circular polarization) component of the signal is attracted to the orthogonal (counter-rotating) polarization component of the pump. This is due to the corresponding negative sign of the third component of the Raman tensor; physically, the change of sign is due to the interchange of the propagation direction, which changes the handedness of the polarization rotation.

When considering now the two open cavity configurations of Fig. 1, we find that the presence of a FBG reduces the laser threshold with respect to the mirrorless case. However, Fig. 3(b) shows that in the open cavity configurations the output laser power is strongly dependent on the value of the pump SOP. Figure 3(b) shows the maximum and minimum values of the output laser power (in either the co or the counter-propagating signal) for scheme B in Fig. 1. The polarization instability of the laser output is due to the cancellation of the circular polarization components of the total intracavity field. In fact, co-propagating and the counter-propagating signals are attracted towards two counter-rotating (when observed in a fixed reference frame) circular polarizations. As a result, the laser output tends to be linearly polarized. Moreover, the lower the circular polarization component of the pump ${S}_{3}^{(p)}$, the more powerful the laser output is.

As can be seen in Fig. 3(c), because of the strong Raman-induced polarization pulling experienced by the co-propagating signal, its circular polarization component ${S}_{3}^{s+}$ closely follows the circular polarization component of the pump ${S}_{3}^{p}$. Whereas Fig. 3(c) shows that the circular polarization component of the counter-propagating signal ${S}_{3}^{s-}$ remains close to zero for $|{S}_{3}^{p}| < 0.7$. The output DOP in the co-propagating signal remains higher than 96% at all times. By using scheme A in Fig. 1, we obtain similar results, however with comparatively slightly lower DOP values, because of the reduced DOP of the counter-propagating signal arriving at the FBG. In this configuration, the SOP of the counter-propagating signal changes with the SOP of the pump: its circular component (${S}_{3}^{s-}$) is inversely proportional to the corresponding component of the pump (${S}_{3}^{p}$). On the other hand, the cancellation of the circular component is evident in the co-propagating signal, that remains always linearly polarized.

### Experimental results and discussion

**Figure 4:**
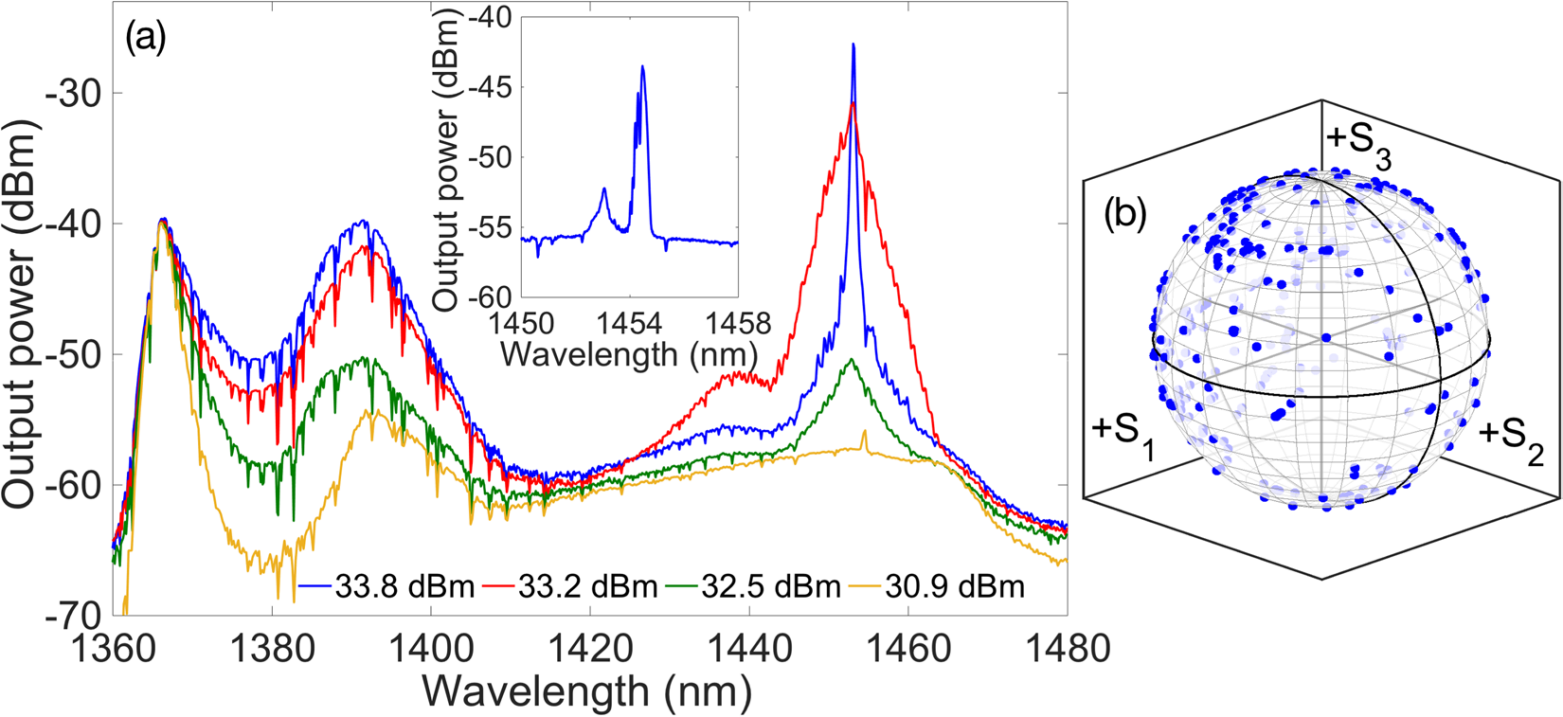
Experimental results for scheme A: (**a**) spectrum of the co-propagating signal output. In the inset, we show the output spectrum of the counter-propagating signal. (**b**) Poincaré sphere display of the variation of the SOP of the 1453 nm co-propagating output signal, as the pump SOP is varied.

Let us focus first on the configuration with the FBG at the pumping end, as in scheme A of Fig. 1. As discussed in the previous simulation section, open cavity configurations are expected to display a lower threshold and improved pumping efficiency with respect to the mirrorless case. The corresponding experimentally observed output spectra are shown in Fig. 4(a). The spectra are shown between 1360 nm and 1480, as no features of interest are observed outside this range. As can be seen, the evolution of the output laser spectrum with pump power can be divided in two different regimes, as far as the nonlinear effects involved in the laser dynamics are concerned. The first regime is dominated by the presence of XPM and parametric scattering, whereas the second regime is governed by the action of Raman scattering. For relatively low pump powers, one observes the generation of a narrow signal at the 1454.5 nm FBG wavelength, sitting on a broad supercontinuum with a peak at 1393 nm. The higher the pump power, the higher the signal power level at the FBG frequency. For pump powers higher than 32 dBm, a new spectral peak appears at 1453 nm, which quickly superimposes over the initial 1454.5 nm peak. The amplitude of the 1453 nm peak grows larger with pump power, up to pump powers close to 33.5 dBm. Next, the effects of Raman scattering become dominant, until lasing begins at 1453 nm. Subsequently, most power below 1440 nm is redistributed under the lasing peak. Note that the lasing wavelength is slightly different from the FBG resonant wavelength: it corresponds to the peak of the Raman gain profile. The presence of a FBG has no influence on the generation of the laser emission, where the feedback is provided by distributed Rayleigh backscattering (i.e., it corresponds to the generation of random distributed feedback lasing). Whenever the open cavity is pumped with a depolarized pump (i.e., without the polarising prism), Raman gain never reaches a sufficient level to prevent strong supercontinuum generation at 1393 nm. As a result, with the open cavity we could not achieve lasing at 1453 nm for injected pump powers up to 35.5 dBm. As a result, we may infer that polarization alignment between the pump and the generated Stokes is critical to achieve an efficient Raman frequency conversion. This polarization alignment is maintained thanks to the low PMD of the spun fiber, which in turn enables the generation of highly polarized random lasing.

For scheme A of Fig. 1, we experimentally found that the co-propagating laser output is not fully polarized: in this configuration the output signal power, its DOP and the associated Raman conversion efficiency strongly depends on the input SOP of the pump. As a matter of fact, by varying the SOP of the pump by means of the PC, we observed that the output laser DOP oscillates between 65% and 95%. Correspondingly, as shown in Fig. 4(b), the SOP of the laser output widely moves across the entire Poincaré sphere. A fully polarized (i.e., with a DOP equal to 100%) laser output cannot be obtained since the lasing signal also includes the contribution from the FBG reflected signal at 1454.5 nm which, according to the simulations, is never fully polarized. Moreover, the observed forward laser output DOP dependence on the pump SOP may be explained as follows. The FBG reflected 1454.5 nm component is mainly attracted to a linear SOP. Hence maximum (minimum) DOP of the total laser output (resulting from the sum of the lasing signal and the signal reflected by the FBG) is obtained when the pump itself is linearly (circularly) polarized. The linear SOP pump, which produces the maximum values of the laser signal DOP, also produces the highest output power.

When measuring the output at the pumping end, no lasing was obtained, owing to the reduced polarization attraction in the counter-propagating signal configuration. As shown in the inset of Fig. 4(a), we observed two spectral peaks at 1453 nm and 1454.5 nm, respectively. The first peak corresponds with the Rayleigh backscattering of the lasing signal, whilst the second peak matches the resonant wavelength of the FBG. Due to the position of the FBG, the power of the 1454.5 nm signal is not sufficient for filtering it out, and measuring its polarization characteristics. Nevertheless, the DOP of the unfiltered signal is less than 30%, which indicates that the 1454.5 nm signal is effectively depolarized.

**Figure 5:**
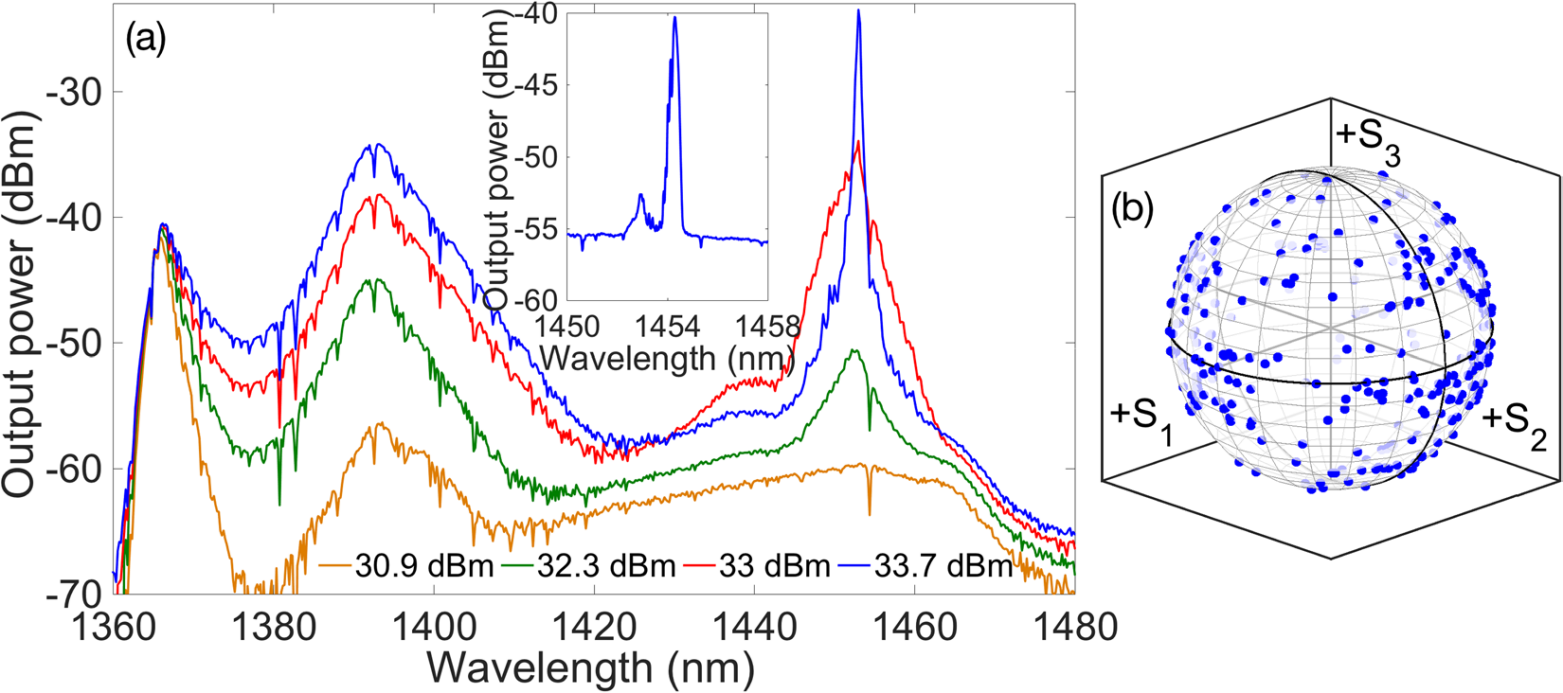
Experimental results for scheme B: (**a**) spectrum of the co-propagating output. In the inset, we show the spectrum of the counter-propagating signal; (**b**) Poincaré sphere display of the variation of the SOP of the 1453 nm co-propagating output signal.

Let us consider now scheme B of Fig. 1, with a FBG placed at the opposite side of the pump. The corresponding experimental results are summarized in Fig. 5. As it can be seen, the co-propagating output spectrum remains almost identical to that obtained with the previous configuration (see Fig. 4(a)). In particular, the output laser power remains largely independent of the position of the FBG, allowing stable output lasing at 1 mW when pumped with a 1 W linear SOP pump. However, the DOP of the 1453 nm signal is slightly higher with scheme B, as it varies now between 75% and 100%. Nevertheless, Fig. 5(b) shows that also with scheme B the SOP of the output co-propagating laser Stokes signal is largely dependent on the pump SOP value: its values almost uniformly cover the Poincaré sphere.

**Figure 6:**
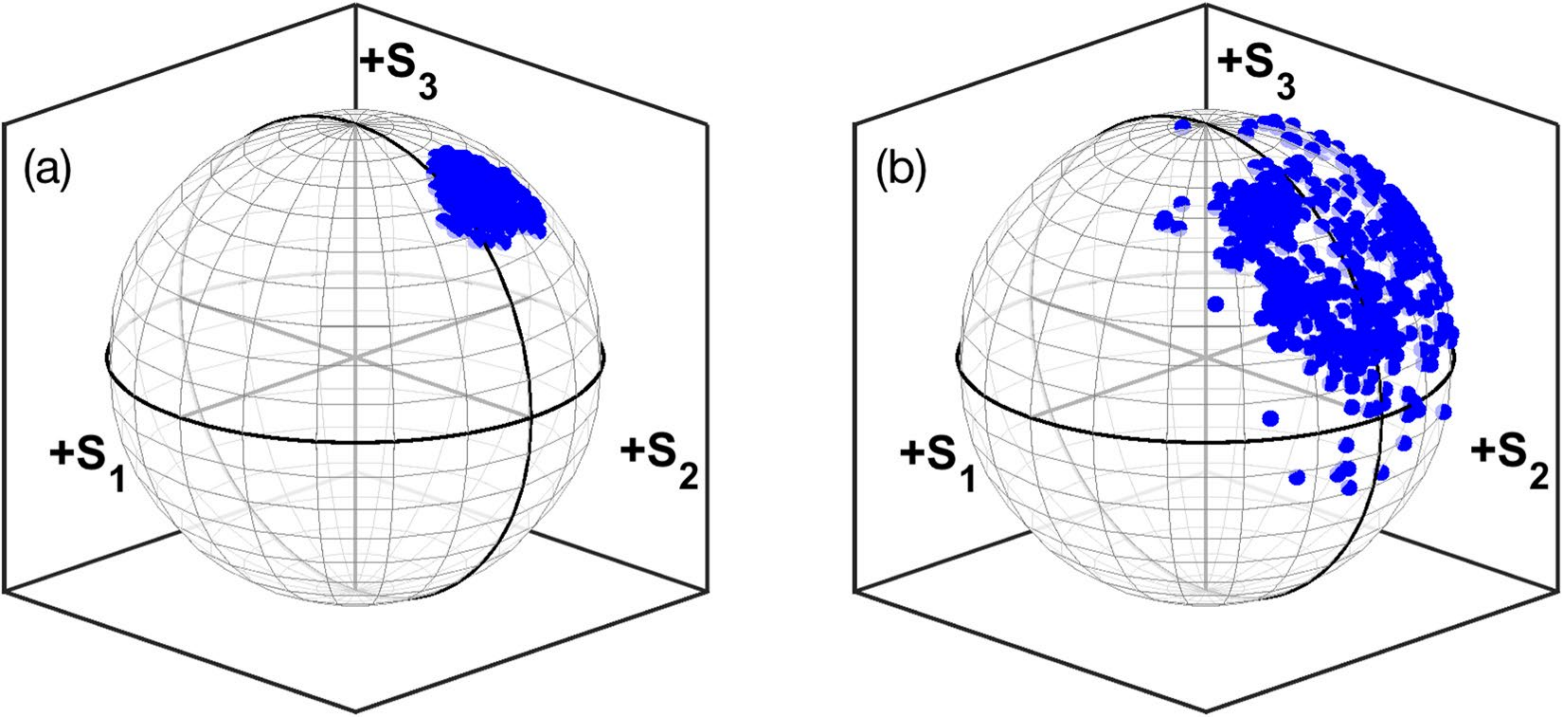
(**a**) Poincaré sphere display of the variation of the SOP of the 1454.5 nm counter-propagating output signal in configuration 2 with a spun fiber; (**b**) As in (**a**), but using a SMF.

The most remarkable differences between schemes 1 and 2 of Fig. 1 are observed in the counter-propagating output signal. Firstly, by comparing the insets of Figs 4(a) and 5(a), we can see that the output power of the 1454.5 nm counter-propagating output Stokes signal is higher with scheme B. This could be expected, given the position of the FBG at the end of the cavity. Next, we found that the output counter-propagating Stokes signal power is slightly dependent on the pump SOP value. Most importantly, we observed that the counter-propagating output Stokes signal DOP remains extremely close to 100% for any value of the pump SOP. In addition, as shown in Fig. 6(a), the output signal maintains a well-defined SOP, which is virtually unaffected by the SOP of the pump. Quite strikingly, an highly polarized counter-propagating signal output is also observed whenever a depolarized pump is employed. These observations suggest the presence of a strong self-attraction of the SOP of the 1454.5 nm Stokes signal, as it occurs in the omnipolarizer^17^. According to the qualitative information provided by the numerical solution of the model of Eqs (1–3), the Stokes signal should indeed preserve a linear SOP, even for a relatively high contribution of circular polarization in the pump. Hence, when observed on the Poincaré sphere, variations of the pump SOP should mostly translate into a displacement on the output Stokes SOP over points close to the equator. Experimental observations show that the Stokes SOP has an important component of clockwise circular polarization, which can be attributed to the presence of discrete polarization rotation elements in the light path, such as the FBG^34^. It is worth noting that output powers were stable in all the considered cases,even if the spun fiber is subject to slight movements, these movements, though, can lead to instantaneous rotations of the output SOP.

**Figure 7:**
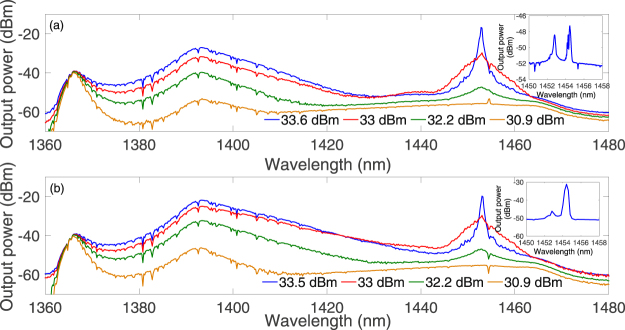
(**a**) Experimental spectrum of the co-propagating output for scheme A using SMF. In the inset, we show the spectrum of the counter-propagating signal; (**b**) Experimental sprectrum of the co-propagating output for scheme B using SMF. In the inset, we show the spectrum of the counter-propagating signal.

In order to appreciate the role of the ultra-low PMD spun fiber in determining the effectiveness of Raman polarization attraction and random lasing, we repeated our experiments by replacing the spun fiber with a standard fiber. We used a 3 km span of SMF-28. SMF has a lower linear loss and a smaller core area, hence a higher Raman gain coefficient, than the spun fiber. Moreover the used SMF span was also slightly longer than the spun fiber span, which could also be expected to contribute to more efficient amplification and lasing, in the absence of any polarization effect. In spite of all of these potential advantages, we observed that, when a highly polarized pump is used, the higher PMD in the SMF leads to an overall reduced Raman gain with respect to spun fiber case. The experimental spectra are shown in Fig. 7.

In fact, we observed that, when using the SMF, a depolarized pump and a FBG at the pumping end, lasing could not be achieved within the available pump powers: instead, a supercontinuum spectrum was generated. On the other hand, with a fully polarized pump and the SMF, lasing at 1453 nm was easily achievable in the co-propagating direction, with a threshold of only 0.2 dBm lower than with the spun fiber. The output DOP varied between 40% and 100%, again depending on the input SOP of the pump.

With an FBG at the far end of the pump and using the SMF, we observed that the counter-propagating Stokes output power at the pumping end was again higher than with the spun fiber, as could be expected, given the extra length and higher gain coefficient of the SMF. However, the output DOP is much reduced when using an SMF, and the corresponding SOP no longer remains fixed as the pump SOP is varied. The DOP of the output Stokes signal varied between 30% and 85%, depending on the configuration of the PC. As shown in Fig. 6(b), the signal SOPs are distributed over a much broader range in the Poincaré sphere than in the spun fiber case, owing to the much higher PMD of the SMF. The output SOP distributions on the Poincaré sphere with either a spun fiber or an SMF can be clearly compared in Fig. 6.

In conclusion, two different configurations of open-cavity Raman fiber lasers relying on low-PMD spun fiber and high-power, polarized CW pumping have been proposed, theoretically studied and experimentally demonstrated and characterized for the first time. The obtained experimental results are in good agreement with the predictions of our numerical simulations, based on a simple theoretical model for the interaction of counter-propagating beams in random distributed feedback fiber lasers. The configuration with the fiber grating at the far end of the pump turns out to be the most efficient, since it displays a lower lasing threshold. Narrow-spectrum lasing in the co-propagating direction with respect to the pump is observed at a wavelength which differs from the central FBG reflection wavelength, but coincides with the Raman gain peak. These characteristics, together with the absence of an identifiable mode structure in the RF output spectrum, provide evidence of efficient random lasing with feedback provided by Rayleigh scattering.

On the other hand, lasing in the counter-propagating output signal was generally less efficient, owing to the reduced efficiency of polarization attraction. Nevertheless, a highly polarized counter-propagating output signal was obtained at the FBG wavelength. Interestingly enough, a high counter-propagating signal DOP could be obtained in this configuration even when using a depolarized pump, which can be attributed to a self-attraction (or omnipolarizer) effect combined with the polarization maintaining properties of the spun fiber. For comparison, experiments were repeated using a comparable length of standard single-mode communications fiber. Although the lasing threshold was slightly reduced in this case, owing to the smaller fiber attenuation and slightly longer fiber length, its value remains comparable to that of the spun fiber. In fact, the latter offers improved polarization-dependent Raman gain, thanks to its much lower birefringence. Additionally, we observed that the DOP of the laser output is considerably reduced when a SMF is used: the corresponding output SOPs are distributed over a much broader area on the Poincaré sphere. Our results demonstrate, for the first time in the context of random distributed feedback fiber Raman lasers, the possibility to achieve controllable polarization-pulling and simultaneous lasing at different wavelengths with different output polarization properties.These results not only contribute to improving our understanding of polarization dynamics in ultralong lasers, but also open the possibility of designing future fiber laser sources that may be adequate for polarization-sensitive amplification or sensing.
